# P-2261. Belatacept-based immunosuppression does not confer increased risk of BK Polyomavirus -DNAemia relative to Tacrolimus-based immunosuppression

**DOI:** 10.1093/ofid/ofae631.2414

**Published:** 2025-01-29

**Authors:** Emily Eichenberger, Wairimu Magua, Geeta Karadkhele, Mohammad Kazem Fallahzadeh, Payaswini Vasanth, Christian P Larsen

**Affiliations:** Emory School of Medicine, Atlanta, Georgia; Emory University, Atlanta, Georgia; Emory University School of Medicine, Atlanta, Georgia, Atlanta, Georgia; Emory University School of Medicine, at, Georgia; Emory University School of Medicine, at, Georgia; Emory University School of Medicine, at, Georgia

## Abstract

**Background:**

BK polyoma virus (BKPyV) DNAemia imparts significant morbidity in kidney transplant recipients (KTR). It can lead to polyomavirus-associated nephropathy (PyVAN), impaired graft function, and graft failure. The effect of belatacept on BK polyoma virus (BKPyV) control remains largely unknown.

**Methods:**

This is a propensity matched retrospective cohort study in adult kidney transplant recipients (KTR) transplanted between 2016-2020 who received a belatacept- versus tacrolimus-based immunosuppression regimen. A multi-state Markov model was used to evaluate BKPyV replication dynamics (BKPyV-dyn). Three BKPyV-dyn states were defined: BKPyV-dyn1 (viral load < 3 log_10_), BKPyV-dyn2 (viral load > 3 log_10_ and < 4 log_10_), and BKPyV-dyn3 (viral load > 4 log_10_).

Estimated total length of stay in a BKPyV replication dynamic state after evolution of the entire Markov process

**Figure:**
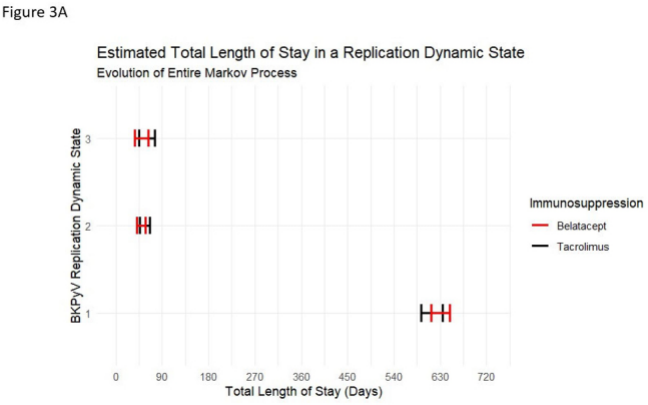

**Results:**

280 KTR on belatacept- and 280 KTR on tacrolimus-based regimens were compared. The probability of transitioning between BKPyV-dyn states and time spent in each state in both groups was comparable. Total duration in BKPyV-dyn-1 was 632.1 days (95%CI 612.1, 648.5) for belatacept vs 615.2 days (95% CI 592.5, 635.8) for tacrolimus, BKPyV-dyn-2 was 49.2 days (95% CI 41.3, 58.4) for belatacept vs 55.6 days (95% CI 46.5, 66.8) for tacrolimus, and BKPyV-dyn-3 was 48.7 days (95% CI 52.3, 79.5) for belatacept vs 60.9 days (95% CI 48.3, 76.4) for tacrolimus (Figure 1). Estimated mean sojourn time did (time spent in a given BKPyV-dyn state prior to transitioning to another state) did not differ by immunosuppression (Figure 2). BKPyV associated nephropathy (PyVAN) occurred in 3.9% in belatacept- and 3.9% tacrolimus-treated KRT (P >0.9).

Estimated mean sojourn time by immunosuppression

**Figure:**
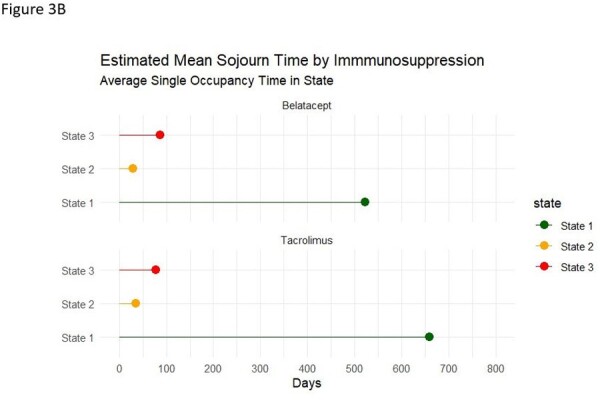

Average single occupancy time in a BKPyV replication dynamic state

**Conclusion:**

Collectively, our results indicate that relative to patients on tacrolimus-based regimens, patients on belatacept-based immunosuppression regimens do not require more frequent BKPyV QNAT monitoring or more aggressive immunosuppression reduction when BKPyV-DNAemia 3 log_10_ or greater is detected.

**Disclosures:**

Christian P. Larsen, MD, PhD, Bristol-Myers Squibb: Advisor/Consultant|CareDx: Advisor/Consultant|Eledon: Advisor/Consultant

